# The Rare Earth Element Lanthanum (La) Accumulates in *Brassica rapa* L. and Affects the Plant Metabolism and Mineral Nutrition

**DOI:** 10.3390/plants14050692

**Published:** 2025-02-24

**Authors:** Cong van Doan, Moez Maghrebi, Noemi Gatti, Giuseppe Mannino, Gianpiero Vigani, Massimo E. Maffei

**Affiliations:** Plant Physiology Unit, Department of Life Sciences and Systems Biology, University of Turin, Via Quarello15/a, 10135 Turin, Italy; moez.maghrebi@unito.it (M.M.); noemi.gatti@unito.it (N.G.); giuseppe.mannino@unito.it (G.M.); gianpiero.vigani@unito.it (G.V.); massimo.maffei@unito.it (M.E.M.)

**Keywords:** lanthanum, *Brassica rapa*, rare earth elements, flavonoids, mineral nutrition, reactive oxygen species, fatty acids, gene expression

## Abstract

Lanthanum (La) is often used in industry and agriculture, leading to its accumulation in natural environments and potential ecological risks. The objective of this study was to examine the effects on the growth, metabolism, and nutrient composition of *Brassica rapa* exposed to at low (1 µM), medium (1 mM), and high (10 mM) La concentrations. We used chemical analytical, molecular, and metabolomic methods and found that high La exposure induced a hormetic effect, triggering both stimulatory and inhibitory responses. La reduced aluminum (Al), cobalt (Co), nickel (Ni), and chromium (Cr) levels at all concentrations, while medium and high doses also decreased phosphorus (P) and iron (Fe). La accumulation in *B. rapa* increased with La levels, affecting metabolic processes by modulating reactive oxygen species (ROS), increasing proline, and reducing total polyphenol content. Flavonoid levels were altered, chlorophyll and carotenoids declined, and non-photochemical quenching increased. Gene expressions related to flavonoid, carotenoid, and chlorophyll metabolism, as well as ion transport, exhibited a dose-dependent modulation. On the contrary, fatty acid composition remained unaffected. Our results indicate that La accumulates in in *B. rapa* and disrupts the plant metabolism. Despite an evident effect on plant productivity, our results also raise concerns about the potential health risks of consuming La-enriched *B. rapa* plants.

## 1. Introduction

Lanthanum (La) is a naturally occurring element belonging to a group of 17 similar metallic elements known as Rare Earth Elements (REEs), found in the Earth’s crust [1]. It is the second most abundant REE, following Cerium (Ce) [2], and is particularly present in soils and waters contaminated by human activities, raising environmental concerns, especially in developing countries [3]. Despite these concerns, La is considered a beneficial element for plant growth [4]. Indeed, La has been utilized as a fertilizer to improve the production of various crops, including tea (*Camellia sinensis*) [5], oat (*Avena sativa*) [6], maize (*Zea mays*) [7], wheat (*Triticum aestivum*) [8], rice (*Oryza sativa*) [9], and some legumes such as *Vigna angularis* L. and *Glycine max* L. [10,11]. The effect of La on plant growth is influenced by its concentration levels. At low concentrations, La promotes the synthesis of chlorophyll, enhances plant growth and development, and improves nutrient uptake [12]. In contrast, higher concentrations of La can inhibit growth [1,13] and disrupt cell division by affecting the levels of chlorophylls, sugars, amino acids, and proteins [14,15]. Additionally, La can inhibit photosynthesis by increasing the production of reactive oxygen species (ROS) and causing damage to chloroplasts [16,17]. Moreover, high levels of La potentially interfere with nutrient absorption in cells by altering potassium and calcium channels and reducing ATP production [18,19].

Plants can acquire La from the soil and, due to its high mobility, it can accumulate in different plant tissues [20]. However, the level of La accumulation is tissue- and plant-species-dependent [1]. Therefore, the presence of La in food through dietary intake has raised concern for human health [21]. In particular, La-based materials used in water treatment pose potential risks to human health and ecosystems [22]. Moreover, sensitivity analysis indicates that underweight individuals are more susceptible to health risks associated with lanthanum concentrations, with food intake playing a significant role in these risks [23]. Lanthanum (La) and similar rare earth elements (REEs) tend to undergo trophic dilution rather than biomagnification [24].

*Brassica rapa*, a member of the Brassicaceae family, is an important food crop and a staple in daily diets. This species includes various vegetable forms, such as Chinese cabbage, pak choi, and turnip, as well as forage and oilseed plants [25]. Research has shown that low doses of La can affect plant growth, mineral uptake, and the production of secondary metabolites in *B. juncea* [26] and *B. chinensis* [27]. In *B. napus*, La has been found to enhance the transport of aluminium in the roots [28]. However, there is limited knowledge regarding the effects of La on *B. rapa*.

The objectives of this study are to evaluate the possibility of La accumulating in *B. rapa* and to assess the impact of La on the development, metabolism, and mineral nutrient content of this important food crop. Here, we show that La accumulation affects the performance of *B. rapa* by altering its metabolism and nutrient composition.

Understanding the dose-dependent effects of La on this important crop will be crucial for understanding the potential implications of the occurrence of elevated La in natural ecosystems, and the implications of La contamination in cultivated areas for food chains and human health.

## 2. Results

### 2.1. Influence of La on B. rapa Plant Growth and Biomass Production

**Figure 1:**
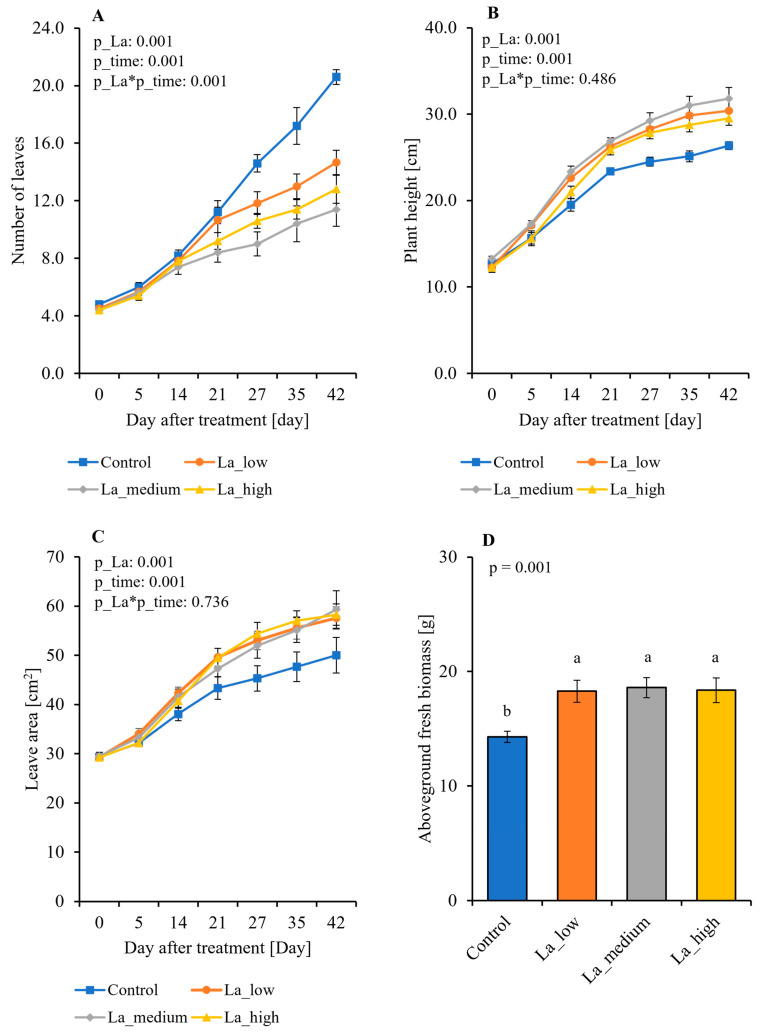
Impact of lanthanum (La) on *Brassica rapa*’s development. (**A**) Number of leaves; (**B**) plant height; (**C**) leaf area; (**D**) aboveground biomass, after 42 days La application; La_low = 1 µM, La_medium = 1 mM, La_high = 10 mM. Mean + SE, n = 5–8. Different letters indicate a statistically significant difference (*p* < 0.05).

To evaluate the effects of La on the shoot development of *B. rapa*, plants were subjected to different increasing concentrations, and three different concentrations of La were selected: low (1 µM), medium (1 mM), and high (10 mM). The growth of *B. rapa* was monitored for 42 days. La-treated plants displayed a significant (*p* < 0.05) decrease in the total leaf number (Figure 1A) and an increase (*p* < 0.05) in both plant height (Figure 1B) and leaf area (Figure 1C) compared to control plants. Furthermore, La-treated plants displayed an increase in aboveground biomass, regardless of La concentration (Figure 1D).

### 2.2. Elevated Concentrations of La Decrease Mineral Ions of B. rapa

To evaluate the effects of increasing concentrations of La on the plant mineral nutrition of *B. rapa*, we analyzed the content of several mineral nutrients using ICP-MS. La content accumulated in *B. rapa* leaves. Lanthanum increased with La administration, rising from 0.52 (±0.17) fold change (FC) at low concentration to 1.64 (±0.24) FC at medium, and reaching up to 40.88 (±5.01) FC at high La concentrations (Table 1).

The application of La reduced the concentrations of Al, Cr, Co, Ni, and Cr (*p* < 0.05) in leaves with respect to the control condition. Additionally, medium and high concentrations of La induced a decrease (*p* < 0.05) in Fe and Mo contents (Table 1) in *B. rapa*. At high La concentration, plants displayed a decrease (*p* < 0.05) in Ca and P content and an increase in K, while the content of the other elements was not affected (*p* > 0.05), with respect to control plants (Table 1). Non-significant differences were found for most of the heavy metals as well as for Mo, Na, Cu, Zn, Mn, and Ti (Table 1).

### 2.3. Lanthanum Modulates ROS Production, Decreases Total Polyphenol Content, Increases Proline Contents and Modulates Flavonoid Production of B. rapa Leaves

**Figure 2:**
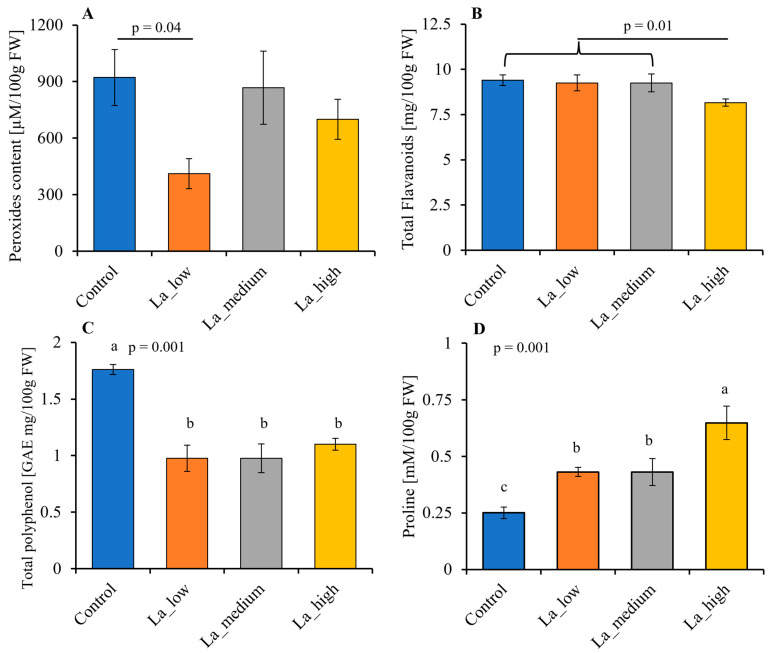
Impact on *B. rapa* peroxides (**A**), total flavanoids (**B**), total polyphenols (**C**), and proline (**D**) leaf content after 42 days of La application at different concentrations. La_low = 1 µM, La_medium = 1 mM, La_high = 10 mM. Metric bars indicate SE, n = 3–6. Different letters indicate a statistically significant difference (*p* < 0.05).

Since La has been found to induce ROS production in some crops, we assessed ROS production and scavenging in *B. rapa* leaves upon La treatment. The production of peroxides in *B. rapa* was unaffected at medium and high La concentrations; however, a decrease (*p* < 0.05) in peroxides content was observed at low La concentrations (Figure 2A).

The total flavonoid content decreased (*p* < 0.05) in the leaves of plants exposed to high concentrations of La (Figure 2B). Furthermore, with respect to controls, the total polyphenol content decreased (*p* < 0.05) in the leaves of plants treated with La, regardless of the concentration used (Figure 2C).

Additionally, La application exhibited a dose-dependent effect on leaf proline content, with higher values observed at high La concentrations (Figure 2D).

**Figure 3:**
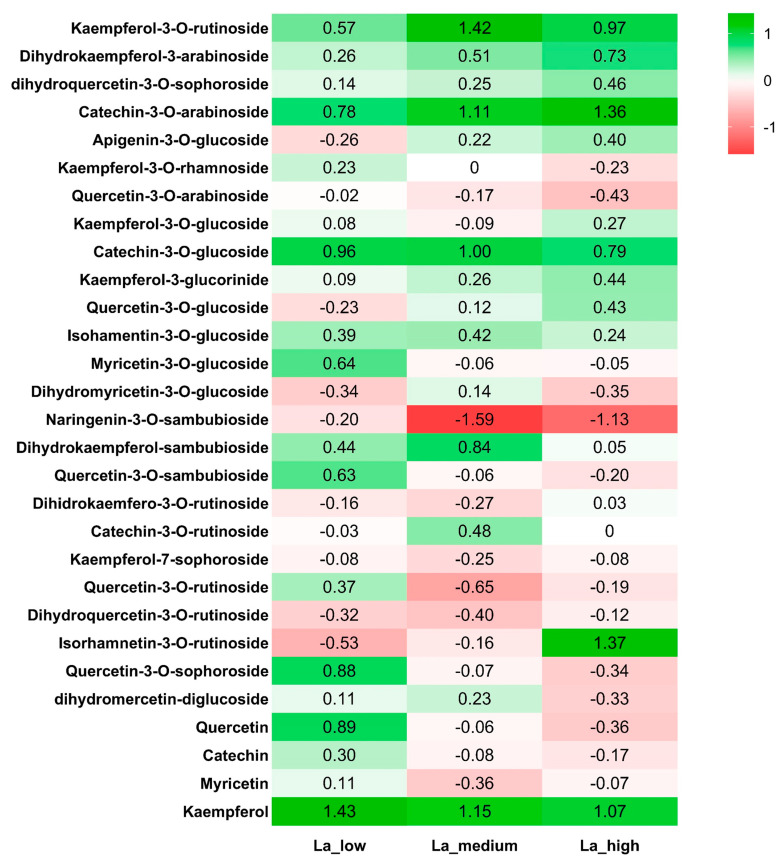
Heatmap of lanthanum (La) effects on *Brassica rapa*’s identified flavonoids profile after 42 days of La application. Values are expressed as log (2) fold change with respect to control (no La application). La_low = 1 µM, La_medium = 1 mM, La_high = 10 mM.

To evaluate the qualitative and quantitative variation in total phenolic compounds, we analyzed the flavonoid content of *B. rapa* using HPLC-MS/MS after exposure to increasing concentrations of La. Lanthanum treatment differentially affected the content of the identified compounds. Specifically, the levels of kaempferol-3-*O*-rutinoside, dihydrokaempferol-3-arabinoside, dihydroquercetin-3-*O*-sophoroside, catechin-3-*O*-arabinoside, catechin-3-*O*-glucoside, kaempferol-3-glucuronide, isorhamnetin-3-*O*-glucoside, and kaempferol increased at all La concentrations. In contrast, apigenin-3-*O*-glucoside and quercetin-3-*O*-glucoside were increased at medium and high concentrations (Figure 3, see also Supplementary Table S1 for raw data). Low concentrations of La also increased the content of kaempferol-3-rhamnoside, myricetin-3-*O*-glucoside, quercetin-3-*O*-sambubioside, quercetin-3-*O*-rutinoside, quercetin-3-*O*-sophoroside, quercetin, catechin, and myricetin (Figure 3 and Supplementary Table S1). On the contrary, all La concentrations resulted in a decrease (*p* < 0.05) in the content of naringenin-3-*O*-sambubioside and dihydroquercetin-3-*O*-rutinoside, while catechin-3-*O*-rutinoside and quercetin-3-*O*-sophoroside decreased only at higher La concentrations (Figure 3 and Supplementary Table S1).

### 2.4. Lanthanum Reduces the Chlorophyll and Carotenoid Contents of B. rapa

**Figure 4:**
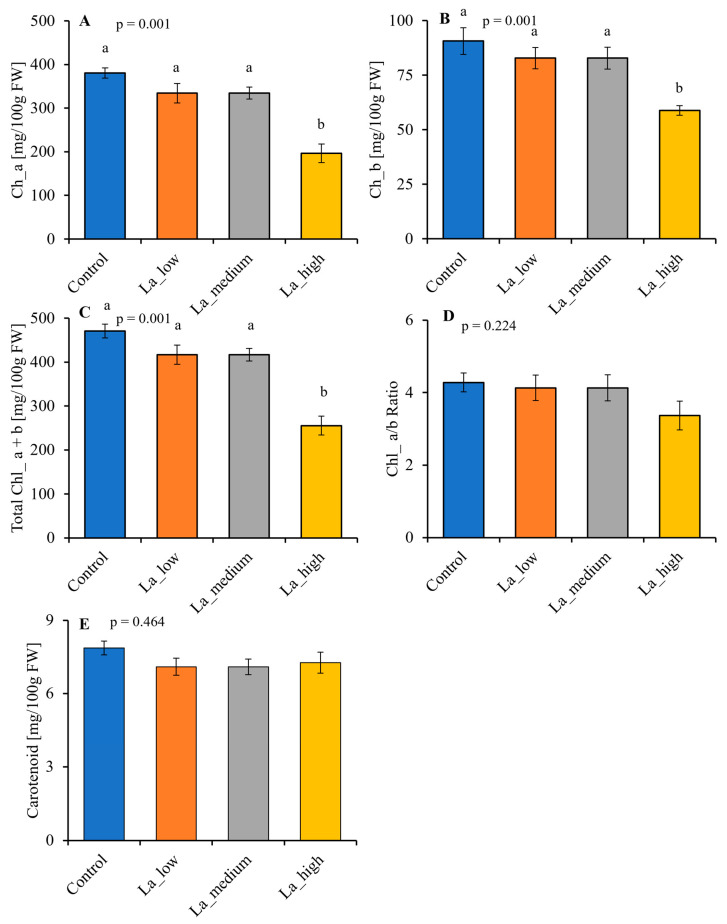
Impact of lanthanum (La) on *Brassica rapa*’s (**A**) chlorophyll a, (**B**) chlorophyll b, (**C**) total chlorophyll, (**D**) chlorophyll a/b ratio, and (**E**) carotenoids content, after 42 days of La application. La_low = 1 µM, La_medium = 1 mM, La_high = 10 mM. Bar indicates mean + SE, n = 3–6. Different letters indicate a statistically significant difference (*p* < 0.05).

With respect to controls, La prompted a dose-dependent effect on chlorophyll content, leading to a reduction (*p* < 0.05) in the levels of chlorophyll a (Figure 4A), chlorophyll b (Figure 4B), and total chlorophyll (Figure 4C), depending on the La concentration, whereas the carotenoid content was reduced independently on the La concentration (Figure 4E). Notably, significant differences (*p* < 0.05) in the chlorophyll a/b ratio were observed only at high La concentrations (Figure 4D).

### 2.5. Lanthanum Increases the Non-Photochemical Quenching of B. rapa

**Figure 5:**
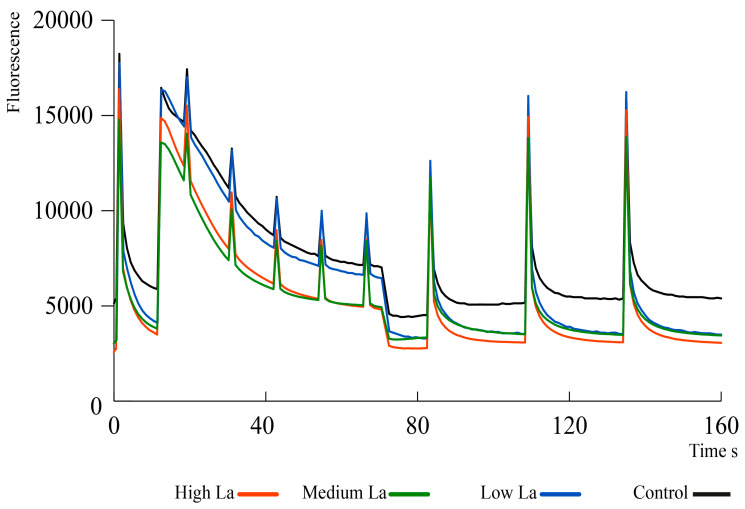
Impact of lanthanum (La) on *Brassica rapa*’s non-photochemical quenching. La_low = 1 µM, La_medium = 1 mM, La_high = 10 mM. n = 3–6.

Leaves of *B. rapa* treated with La showed an increase in non-photochemical quenching (NPQ) at medium and high La concentrations compared to control plants (Figure 5). In particular, an increased photochemical quenching (Qp), or quantum efficiency, as well as an increased quantum yield (Qy) under actinic light was found with respect to controls (see Supplementary Figure S1A). Additionally, the fluorescence kinetics were not affected by La treatments compared with control plants, as revealed by OJIP determination in dark-adapted leaves of *B. rapa* plants (see Supplementary Figure S1B).

### 2.6. Lanthanum Does Not Affect the Fatty Acid Content of B. rapa

The analysis of *B. rapa* exposed to increased concentrations of La allowed the identification of several saturated and unsaturated fatty acids (FA). The major compound was linolenic acid, followed by palmitic and palmitoleic acid (Supplementary Table S2). No significant differences were found between control plants and those treated with La.

### 2.7. Lanthanum Modulates the Expression of Some Genes Involved in B. rapa Metabolism and Ion Transporters

**Figure 6:**
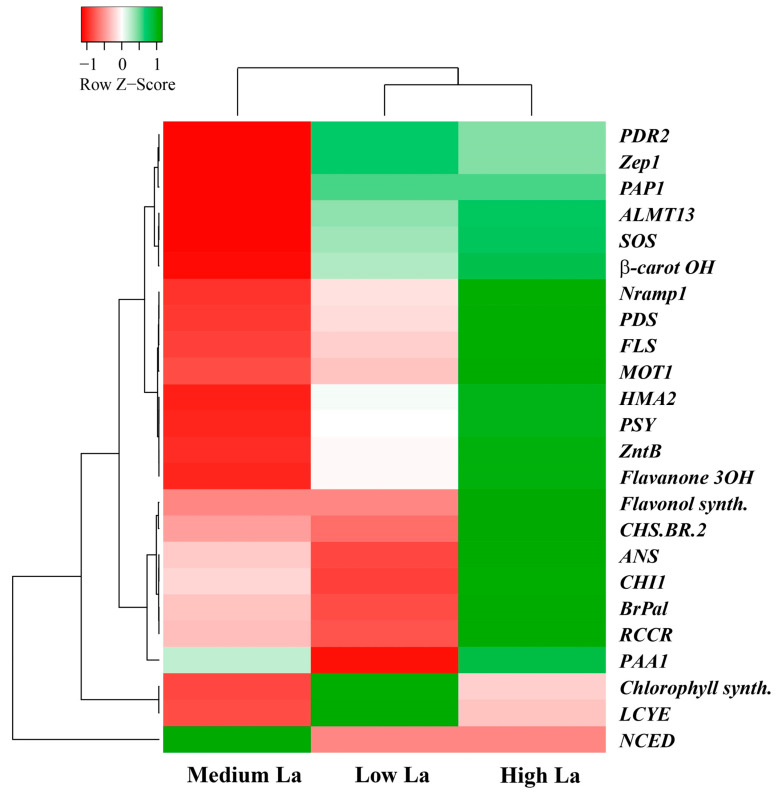
Heatmap form gene expression data of Supplementary Table S3 by using Pearson distance and average linkage clustering methods. The full names of the genes are reported in Table S3. La_low = 1 µM, La_medium = 1 mM, La_high = 10 mM.

To investigate the responses of plants to different La concentrations at the transcriptional level, we conducted qPCR analysis on several genes involved in carotenoid, flavonoid, and chlorophyll synthesis, as well as ion transport. The Log2 fold change of gene expression between La treatment and control plants is reported in Supplementary Table S3. A heatmap generated from the data of Supplementary Table S3, using Pearson distance and average linkage clustering methods, is shown in Figure 6. Generally, a close correlation was found between low and high La concentrations. High concentrations of La resulted in the upregulation of most genes, except two genes involved in the carotenoid metabolism, 9-cis-epoxy carotenoid dioxygenase (*NCED*, LOC103870025) and lycopene epsilon cyclase (*LCYE*, LOC103856778), along with chlorophyll synthase (LOC103841124), which were downregulated. These genes were distinctively clustered from the other genes. Notably, an almost opposite trend was observed in *B. rapa* plants exposed to medium La concentrations, while an intermediate effect between middle and high concentrations was observed in plants treated with a low La concentration (Figure 6).

Similar upregulation (between low and high La concentrations) was found for genes related to carotenoid metabolisms (*Zep1* and beta-carotene 3-hydroxylase 1), as well as for Mn, Na, and Al transporters (*PDR2*, *SOS*, and *ALMT13*) and phenolic compounds (*PAP1*). Conversely, a similar downregulation was found for genes involved in carotenoid synthesis (*NCED*), while an opposite trend (between low and high La treatment) was found for genes coding for proteins involved in (i) phenolic metabolism (putative inactive flavonol synthase 2, *CHS-BR-2*, *CHI1*, *ANS*, and *BrPal*), (ii) chlorophyll metabolism (*RCCR*, chlorophyll synthase), (iii) Cu transporter *(PAA1*), and (iv) carotenoid synthesis (*LCYE*). Between high and medium La concentrations, similar upregulation was found for a gene coding for plastid lipid-associated protein 1 (*PAP1*), a gene coding for chlorophyll synthase, and lycopene epsilon cyclase (*LCYE*).

## 3. Discussion

Although lanthanum (La) has been used as a fertilizer to enhance crop production [29,30], our results show that elevated concentrations of La can significantly affect the growth, metabolism, and nutrient mineral content of *B. rapa*. The effects of La are dose-dependent. Specifically, our findings indicate that applying La increases aboveground fresh biomass, which aligns with its reported use as a fertilizer to improve crop yield. However, La application also reduces the number of leaves, consistent with previously reported inhibitory effects of La on plant growth [31].

The inhibitory effect on plant growth is a consequence of reduced primary metabolism, and fewer leaves leads to a reduced photosynthetic capacity. Photosynthesis has been demonstrated to be affected by La [16], primarily due to the ability of La to accumulate in chloroplasts [32]. Our study showed that high concentrations of La decreased the chlorophyll content in *B. rapa*, as reported in other species [33]. In contrast, the effect of La on carotenoid levels remains relatively stable, suggesting that La might selectively affect chlorophyll biosynthesis [16]. These effects were correlated with an increase in NPQ in *B. rapa* upon La treatment, as found in *Desmodesmus quadricauda* [34], although the quantum yield was not affected. Our work suggests that the photochemistry of *B. rapa* is sensitive to La accumulation. NPQ plays a role in dissipating surplus light energy, thereby preventing the formation of reactive oxygen species (ROS) [35]. Therefore, an increase in NPQ should correlate with reduced ROS production. Accordingly, *B. rapa* decreased peroxides at low La concentrations, suggesting that the increased NPQ effectively mitigated oxidative damage. However, at higher La concentrations, peroxide production was not affected compared to control plants, indicating a threshold beyond which La’s toxic effects may overcome the plant’s protective mechanisms [16]. Furthermore, plants employ other mechanisms to mitigate the deleterious effects of ROS. For instance, phenolic compounds are known to be increased by La, as found in *Helianthus annuus*, *Brassica juncea*, and *Hypericum perforatum* [26,36,37]. The phenolic compounds in *B. rapa* have been widely studied [38]. Our study identified 29 flavonoids within this species, including glycosylated and aglycone forms of catechin, quercetin, kaempferol, myricetin, naringenin, and isorhamnetin, which aligns with the literature data [25,39,40]. Notably, the presence of these flavonoids correlates with the plant’s ability to manage oxidative stress. In *B. rapa* treated with low La concentrations, a significant reduction of the total flavonoid content was found only at the highest La concentration; however, that total polyphenol content was decreased at all La levels under study. This discrepancy is mainly due to the different methods for quantifying phenolic compounds. For this reason, we deepen our analysis by separating the different *B. rapa* polyphenols upon La treatment. A positive correlation was found between the decreased peroxide levels and the increased content of specific flavonoids, particularly catechin-3-*O*-glucoside, catechin-3-*O*-arabinoside, quercetin, and kaempferol. On the other hand, higher La concentrations, which led to increased NPQ, were also associated with increased levels of isorhamnetin-3-*O*-rutinoside. Flavonoids are widely recognized natural antioxidants in plants [41], and our results show that increased La concentrations prompt an increase in scavenging flavonoid levels in *B. rapa*, likely to sustain the oxidative stress caused by La. The upregulation of most genes involved in flavonoid biosynthesis at high La concentrations further supported the hypothesis that these compounds are critical for sustaining oxidative stress responses in *B. rapa*. In summary, the interplay between La treatment and phenolic compound synthesis illustrates a vital adaptive mechanism in plants for mitigating oxidative damage caused by ROS.

The nutrient content is a crucial parameter, and the application of La has been observed to affect the uptake and accumulation of minerals in several plant species [11,42,43]. Besides the soil chemical composition, both controls and treated plants used the same soil nutrients. However, La has been found to decrease the uptake of *B. rapa* essential nutrient elements, as found in in crops like maize, mung bean, and rice [44]. Moreover, La exerts a dose-dependent effect on specific mineral elements [10]. In general, La decreased the content of Al, Cr, and Co in *B. rapa*, while high concentrations of La also led to a decrease in the P content, in agreement with the literature data [1]. The reduction in these elements is concerning given the toxicity of Al and Cr at low concentrations. The aluminum cation (Al^3+^) is known to be toxic to many plants even at very low concentrations [45], while chromium (Cr) is a toxic element that triggers the production of ROS via Fenton and Haber–Weiss reactions in plants [46]. Cobalt (Co) and Al are beneficial for plant growth at a low concentration, but high concentrations have inhibitory effects [47,48]. Therefore, the La-dependent decrease in Co content in leaves observed in our study might correlate with the decreased plant productivity. Similarly, the decreased level of P observed in plants treated with high La concentrations is critical since phosphorus is a key component of several plant metabolites and is involved in various metabolic processes. A deficiency in P can lead to increased production of phenolic compounds, which are often associated with stress responses as found in many plants [49]. This suggests that the reduced P level in *B. rapa* could be linked to increased levels of some observed flavonoids, indicating a stress response mechanism. In general, the imbalance in ion homeostasis resulting from La exposure can disrupt cellular functions, particularly through dysfunctional ion exchanges across cell membranes, affecting lipid composition [50]. However, the lipidomic profile was not affected by La treatment, suggesting that while La influences ion balance and nutrient uptake, its effect on lipid membranes is limited.

Although the content of mineral elements was not affected or decreased (Al, Co, Ni, Mo, and P), most genes involved in ion transport considered in this work were upregulated in response to high La concentrations. This suggests that La affects the level of some mineral ions content and induces nutrient transport in *B. rapa*. The gene coding for Nramp1 (Natural Resistance Associated Macrophage Protein 1), which is involved in the transport of divalent metal ions such as iron and manganese, was upregulated. Its activation has been associated with the plant’s ability to manage excess metal ions, thereby improving metal ion homeostasis [51,52].

In experimental conditions, *B. rapa* exposed to high La levels exhibited a significant increase in the expression of *HMA2*. It is known that *HMA2* is involved in the transport of several elements including Cd and Zn [53,54]. The upregulation of *HMA2*, along with that of other metal transporters like *ZntB*, (a gene involved in Zn homeostasis), *MOT1* (involved in Mo homeostasis), *PAA1* (involved in Cu homeostasis), and *PDR1* (involved in Mn homeostasis), enhances the transport of other metals upon high-La treatment. Indeed, the upregulation of these genes in response to high La concentrations suggests a coordinated response to maintain metals homeostasis under La treatment, and at the same time, it sustains plant micronutrients transport mechanisms to counteract potential disruptions caused by La contamination in *B. rapa*. The *B. rapa* contents of Mn, Zn, and Cu were not affected by La treatment. While primarily associated with zinc transport, ZntB may also facilitate the transport of other metal ions, which is significant in environments where multiple metal ions are present, allowing *B. rapa* to adaptively respond to varying metal ion concentrations [55]. In *B. rapa*, *MOT1* (which is implicated in the Mo uptake in plants [56]) was upregulated in response to high concentrations of La. Mo is a crucial cofactor for important enzymes such as nitrate reductase (NR), sulfide reductase, aldehyde oxidase, and xanthine dehydrogenase [57]. The observed low Mo content in leaves indicated that high La concentration may disrupt Mo homeostasis in plants, potentially impairing the activity of Mo-dependent enzymes. Likely, the impairment of N assimilation might contribute to the observed decreased growth in *B. rapa* treated with high La. *ALMT13* (belonging to the Al-activated malate transporter—ALMT—family) was upregulated in response to high concentrations of La. *ALMT* are involved in the transport of malate and other organic acids that might act as metal-chelating agents [58,59]. In *B. rapa*, exposure to La led to the upregulation of SOS (Salt Overly Sensitive). Studies have shown that under stress conditions, specific genes associated with the SOS response are upregulated, enhancing the plant’s ability to cope with toxic metal accumulation [60,61]. Similarly, *PAA1*, which is implicated in copper ion homeostasis, was upregulated in *B. rapa* in response to high concentrations of La. Such results indicate an impact of La on metal homeostasis by altering the content of some metals (Ni, Mo, and Co) and maintaining the contents of others (Mn, Fe, Cu, and Zn) by upregulating those genes involved in their homeostasis. Such observations suggest that *B. rapa* plants respond to La accumulation by limiting the disruption of intracellular metal homeostasis, which is a prerequisite for cellular redox balance [62]. Accordingly, the synthesis of antioxidant compounds and the induction of detoxification processes indicate that La accumulation triggers mechanisms aimed at restoring redox balance within the cell. The findings suggest that while high concentrations of La do not significantly alter the content of certain minerals like Mn, Zn, and Cu, they do induce a complex regulatory response involving multiple genes responsible for ion transport. This adaptive mechanism allows *B. rapa* to mitigate potential disruptions caused by La contamination while maintaining essential nutrient transport systems necessary for growth and development.

## 4. Materials and Methods

### 4.1. Plant and Chemicals

Seeds of *Brassica rapa* L. were purchased from Deine Gartenwelt, Germany (https://www.deinegartenwelt.de/, accessed on 1 March 2024). Before sowing, the seeds were surface sterilized for 2 min with 90% ethanol and then washed five times with sterile deionized water. The *B. rapa* plants were grown in pots (7 cm × 7.5 cm × 10 cm) containing 100 g of soil (Klasmann-Deilmann GmbH, Geeste, Germany). They were grown under a light intensity of 160 μmol m^−2^ s^−1^ provided by a tunable LED lighting system source (Led Ceiling Light 150 cm 72 W, 7200 LM Neon, ANMECS, Italy) at a temperature of 22 °C (±1.5 °C) with a 16/8 h light/dark photoperiod. La oxide (La_2_O_3_) was purchased from Avantor (https://it.vwr.com/store/, Italy). A suspension of La was prepared in Millipore water, stirred for 5 min to avoid aggregation, and sonicated for 30 min before application. The pH of each suspension was adjusted to 5.6 using a 1.0 M NaOH solution [63].

Leaf surface area was measured by photographing the leaves and calculating their area using Image J v1.54g. We conducted multiple measurements at days 5, 14, 21, 27, 35, and 42 to capture the developmental changes in leaf area over time. Each measurement followed the same procedure to minimize variability and ensure the reliability of the data.

### 4.2. Plant Growth Conditions and Treatments

In all our experiments, plants were not fertilized to avoid possible contamination. To preliminarily assess the effect of La on the germination of *B. rapa*, a range of La concentrations were tested: 0; 0.1; 0.5; 1; 5; 10; 50; 500; 1000 µM. Twenty seeds of *B. rapa* were placed on a Petri dish plate (90 mm diameter) with tissue paper containing 5 mL of La suspension. The germination rate, root, and shoot development were recorded after 3 days. Based on these preliminary results (Supplementary Figure S2), we selected three La concentrations for further experiments: low (1 µM), medium (1 mM), and high (10 mM). Pot experiments were conducted using 21-day-old *B. rapa* plants, which were treated with 10 mL of La suspension, while control plants were watered with distilled water. The experimental design was completely randomized, with three concentrations and six biological replicates, with each replicate consisting of a single plant/pot. We used Traysubstrat Soil (Klasmann-Deilmann GmbH, Geeste, Germany), which is a blend of white and frozen-through black sphagnum peat containing wood fiber (GreenFibre), perlite, clay, and coir. The substrate was uniformly graded, specially screened for modular systems, and included a wetting agent to ensure rapid and even water absorption. The soil does not contain La. The development of *B. rapa* was monitored by measuring the number of leaves, plant height, and leaf area at 5, 14, 21, 27, 35, and 42 days after La application. At 42 days post-application, the leaves were collected, frozen in liquid nitrogen, and stored at −80 °C for further analysis (Supplementary Figure S3).

### 4.3. Chlorophyll, and Carotenoid Analysis

Chlorophylls (Chl) and carotenoids (Car) were extracted and quantified according to [64], with minor modifications. Briefly, 100 mg of ground leaves were extracted with 95% ethanol and the Chl a, Chl b, and Car contents were measured with a UV1280 spectrophotometer (Shimadzu, Kyoto, Japan) at 664 nm, 649 nm, and 470 nm, respectively.

### 4.4. Fatty Acid Methyl Esters Analysis

Fatty acid methyl esters (FAME) were obtained by esterification of *B. rapa* powdered leaves using Boron trifluoride (BF3, 10% *w*/*v* in methanol), with the addition of 50 μg of heptadecanoic acid (C17:0) (Sigma, USA) as an internal standard. Samples were vortexed for 15 s and then incubated for 45 min at 85 °C. Next, 0.5 mL of hexane was added and vortexed for 15 s. Subsequently, 0.5 mL of miliQ water was added and vortexed for an additional 15 s. The mixture was then centrifuged at 12,000× *g* for 15 min at room temperature, and the upper phase containing FAME and hexane was collected. The identification and quantification of FAME were performed using gas chromatography-mass spectrometry (GC-MS) (5975T, Agilent Technologies, Santa Clara, CA, USA) and gas chromatography with flame ionization detector (GC-FID) (GC-2010 Plus, Shimadzu, Kyoto, Japan), respectively, as previously reported [65].

### 4.5. Total Phenolic, Flavonoid and Proline Content

The total phenolic content was determined by the reduction of phosphotungstic-phosphomolybdic acid (Folin–Ciocalteu’s reagent) to blue pigments in an alkaline solution, as previously described [66]. The total flavonoid content was determined by spectrophotometric measurement at 510 nm using aluminum chloride (AlCl_3_), following the method described by reference [67]. Additionally, total proline was determined by spectrophotometric measurement at 510 nm using a ninhydrin solution following the method described by reference [68].

### 4.6. Chemical Characterization of Flavonoids by HPLC-DA-ESI-MS/MS

The identification of *B. rapa* flavonoids was carried out by using HPLC coupled with mass spectrometry. An Agilent Technologies system (1200 HPLC, Agilent Technologies, Santa Clara, CA, USA) was coupled to a diode array detector (DAD) (Agilent Technologies 1200, Santa Clara, CA, USA) and to tandem mass spectrometry (MS/MS, Bruker Daltonics, HB, Germany). This setup, operated in multiple reaction monitoring (MRM) scan type and with electrospray ionization (ESI) source, was used for the separation and identification of compounds as recently reported [69,70]. The limit of detection (LOD) and quantification (LOQ) were calculated by reference [71].

### 4.7. Determination of Total Peroxides

The quantitative content of peroxides was measured using a PEROXsay Assay Kit (G-Biosciences, St. Louis, MO, USA). Fifty mg of freshly ground leaf material was added to one mL of Milli-Q water as previously described [67].

### 4.8. Mineral Element Analysis

Thirty milligrams of dried leaf samples were then mineralized at 225 °C by using 69% nitric acid (HNO_3_). The mineralized material was diluted 1:100 in Milli-Q water and concentrations of mineral elements were subsequently measured using inductively coupled plasma–mass spectrometry (ICP-MS) (BRUKER Aurora-M90 ICP-MS), as previously reported by references [72,73,74]. Supplementary Table S5 shows the raw data of ICP-MS analyses.

### 4.9. Chlorophyll a Fluorescence Kinetics

Chlorophyll fluorescence parameters including F0, Fj, Fi, Fm, Fv, Sm, QY, NPQ, and OJIP were measured using an FP100 fluorometer (Photon Systems Instruments, Drásov, Czech Republic) as previously reported by reference [67].

### 4.10. RNA Preparation, cDNA Cloning, and qRT-PCR Assays

Total RNA was isolated and purified from *B. rapa* leaves using Peqlab PeqGOLD TriFast reagent (VWR Avantor, Radnor, PA, USA). A BioSpec-nano spectrophotometer (Shimadzu, Kyoto, Japan) was used to quantify the extracted RNA. For cDNA synthesis, 500 ng of total RNA was used to synthesize cDNA using qScript Ultra Supermix (Quantabio, Beverly, MA, USA), according to the manufacturer’s instructions. Quantitative Real-Time Polymerase Chain Reaction (qRT-PCR) analysis was performed using QuantStudio 3 Real-Time PCR System (Applied Biosystems, Foster City, CA, USA) in a reaction mixture containing Perfecta SYBR Green Fastmix (Quantabio, Beverly, MA, USA) with ROX as an internal loading standard. A 10 µL mixture consisting of 5 µL 2X Perfecta SYBR Green Fastmix qPCR Master Mix, 0.25 µL cDNA, and 0.01 nmol primers (Integrated DNA Technologies, Coralville, IA, USA) was used. The relative transcript level of each gene was calculated by the ΔΔCt method using the expression of the GADPH gene as a reference. Primers used for qRT-PCR were designed using the Primer3 version 4.1.0 (https://primer3.ut.ee/) software and are reported in Supplementary Table S4.

### 4.11. Statistical Analysis

Each statistical analysis included at least three biological replicates. The data are presented as mean values with fold changes relative to the control, and error bars represent the standard error (SE). To assess the significance of differences between treatments, one-way ANOVA was conducted, followed by the Tukey test for post hoc comparisons. Software SPSS ver. 29 was used.

## 5. Conclusions

In summary, this work revealed the multifaceted effects of lanthanum (La) on plant physiology, particularly regarding the photosynthesis, metabolism, and nutrient status of *B. rapa*. High La concentrations showed a hormetic effect, stimulating certain physiological processes while simultaneously inhibiting others. Elevated levels of La were found to impair photosynthesis and disrupt nutrient uptake mechanisms. Such disruptions can lead to oxidative stress responses, which may compromise plant vitality. In addition, the plant’s ability to upregulate specific genes related to metal ion transport reflects an adaptive strategy to maintain homeostasis in the presence of environmental stressors like La accumulation.

Lanthanum accumulation in crop tissues, such as *B. rapa*, raises significant concerns for ecology, the food chain, and ultimately human health. Therefore, understanding and assessing La accumulation in plants is crucial, particularly due to the rising levels of La in the environment. The novelty of this work is the relationship between the accumulation of La in the leaves of *B. rapa* that demonstrates this crop’s ability to uptake La from the soil and efficiently transfer and accumulate it within its leaf tissue. This finding is consistent with research in other crops, highlighting a potential widespread issue of La uptake and accumulation in food crops. Importantly, our results can be used to assess the potential risk of La presence in foods for human nutrition and human health. Given these potential risks, further investigation into the mechanisms and consequences of La accumulation in *B. rapa* and other crops is of paramount importance.

## Figures and Tables

**Table 1:** Content fold change of treatments vs. controls of several mineral nutrients analyzed by ICP-MS in *B. rapa* leaves exposed to different La concentrations. (±standard error). Boldface numbers indicate significant variation with respect to the control expressed at different levels of significance: * = *p* < 0.05; ** = *p* <0.01; *** = *p* <0.001; ns = *p* > 0.05; x = *p* < 0.1.

Mineral Element	Lanthanum Concentration		
	Low (1 µM)	Medium (1 mM)	High (10 mM)
Al ***	**0.58 ± 0.02**	**0.54 ± 0.03**	**0.46 ± 0.01**
Cr *	**0.76 ± 0.13**	**0.56 ± 0.04**	**0.58 ± 0.03**
Mo **^x^**	1.02 ± 0.19	0.65 ± 0.09	0.59 ± 0.16
Co **	**0.62 ± 0.04**	**0.65 ± 0.04**	**0.62 ± 0.11**
Ni **	**0.81 ± 0.02**	**0.67 ± 0.05**	**0.63 ± 0.1**
P ***	**0.8 ± 0.03**	**1.23 ± 0.07**	**0.64 ± 0.04**
Fe ***	**1.02 ± 0.02**	**0.88 ± 0.05**	**0.79 ± 0.02**
Se *	**0.84 ± 0.09**	**1.12 ± 0.07**	**0.8 ± 0.05**
Na ^ns^	0.86 ± 0.07	0.72 ± 0.1	0.87 ± 0.11
Ca **	**0.86 ± 0.04**	**1.06 ± 0.04**	**0.88 ± 0.03**
Mg *	**0.9 ± 0.04**	**0.99 ± 0.03**	**0.9 ± 0.02**
Cu ^x^	1.03 ± 0.05	0.96 ± 0.01	0.9 ± 0.04
Cd ^ns^	0.83 ± 0.06	1 ± 0.07	0.93 ± 0.08
Zn ^ns^	1.02 ± 0.08	0.9 ± 0.06	1.01 ± 0.06
Pb ^ns^	1.01 ± 0.07	0.94 ± 0.08	1.07 ± 0.09
As ^ns^	1.05 ± 0.18	0.81 ± 0.12	1.08 ± 0.13
Mn ^x^	1.08 ± 0.04	1.23 ± 0.06	1.15 ± 0.09
K **	**0.84 ± 0.07**	**0.9 ± 0.02**	**1.2 ± 0.13**
Tl ^x^	0.93 ± 0.14	0.95 ± 0.07	1.26 ± 0.14
La ***	**0.52 ± 0.17**	**1.64 ± 0.24**	**40.88 ± 5.01**

## Data Availability

Data will be made available on request.

## Supplementary Materials

The following supporting information can be downloaded at: https://www.mdpi.com/article/10.3390/plants14050692/s1. Table S1. Quantitative and qualitative determination of flavonoids extracted from *B. rapa* leaves of plants exposed to increasing La concentrations. Data are expressed as mg g^−1^ fr.wt (standard deviation). Table S2. Fatty acid composition of *B. rapa* leaves exposed to increasing La concentrations. Values are expressed as mg g^−1^ fr.wt. Table S3. Expression (Log2 fold change) of selected genes in *Brassica rapa* after 42 days of exposure to La. The Log2 (fold in change) means comparative gene expression levels between La treatment and control. ns: *p* > 0.05; 0.05 < *p* < 0.1; *: *p* < 0.05, **: *p* < 0.01, ***: *p* < 0.001. Different shade colors correspond to the heatmap colors of Figure 6. Table S4. Primers for genes used in this work. Table S5. Raw data from ICP-MS analyses. Figure S1. (A) Non-photochemical quenching parameters. (B) OJIP parameters calculated in dark-adapted leaves of *B. rapa* plants. Figure S2. Impact of lanthanum (La) on germination of *Brassica rapa*. (A) Length of shoot and (B) root after 3 days germination. Figure S3. The photograph of all treatment groups.
