# Supplementary material for: The Rare Earth Element Lanthanum (La) Accumulates in Brassica rapa L. and Affects the Plant Metabolism and Mineral Nutrition
Source: Plants (Basel). 2025 Feb 24;14(5):692. doi: 10.3390/plants14050692 (PMC11901600; doi:10.3390/plants14050692)

Supplementary Figure S1.

A. Non photochemical quenching parameters

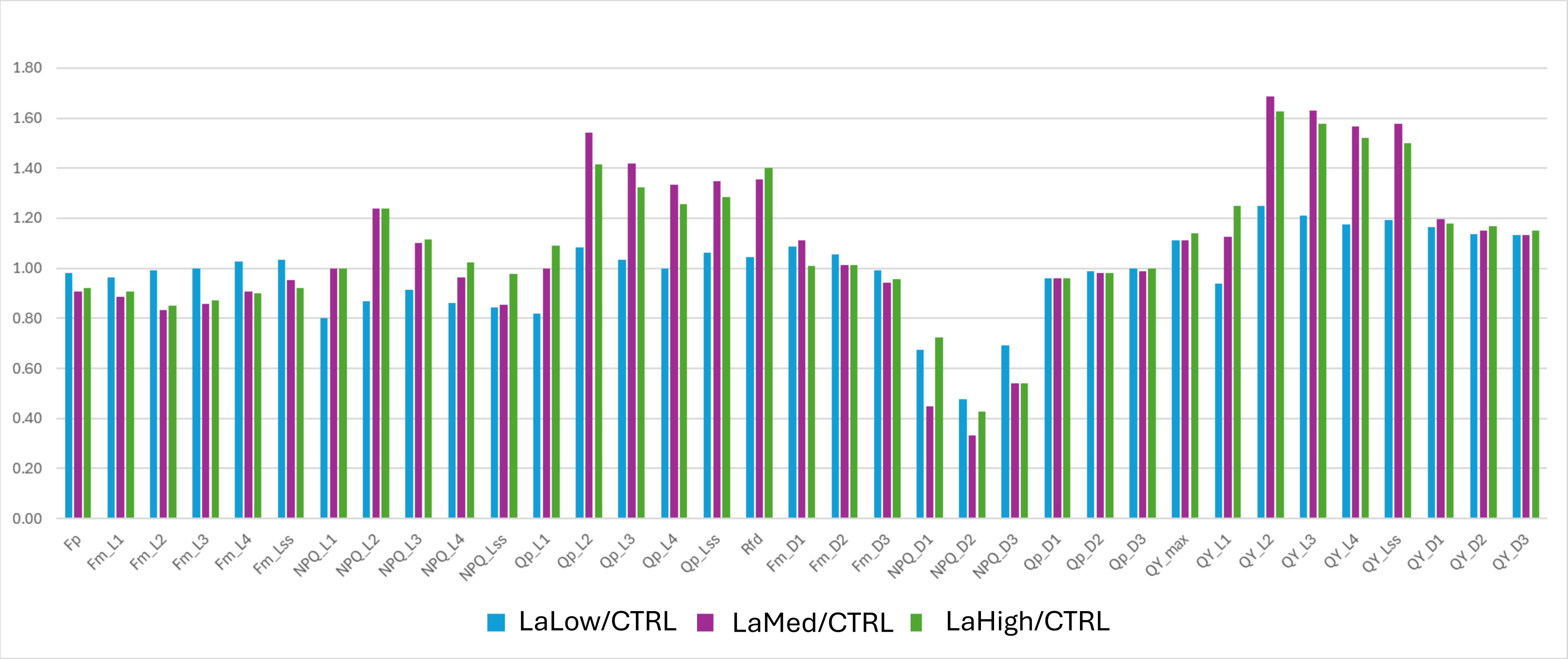

B. OJIP parameters calculated in dark-adapted leaves of *B.rapa* plants

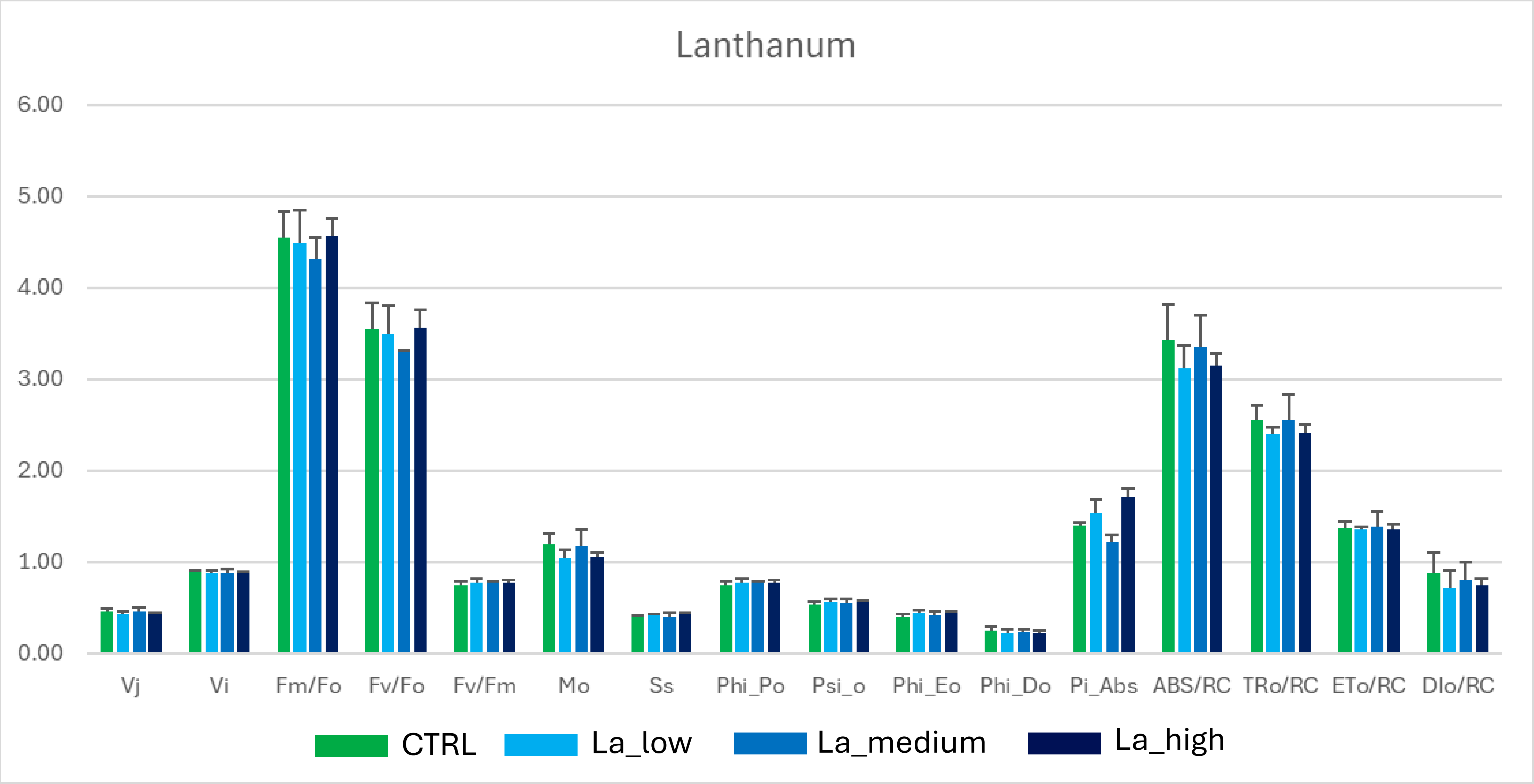

Supplement: Supplementary file 1 [file plants-14-00692-s001.zip › Supplementary Figure S1.pdf]
