# Supplementary material for: The Rare Earth Element Lanthanum (La) Accumulates in Brassica rapa L. and Affects the Plant Metabolism and Mineral Nutrition
Source: Plants (Basel). 2025 Feb 24;14(5):692. doi: 10.3390/plants14050692 (PMC11901600; doi:10.3390/plants14050692)

Supplementary Figure S2. Impact of Lanthanum (La) on germination of *Brassica rapa*. A. Length of Shoot and B. Root after 3 days germincation)

A

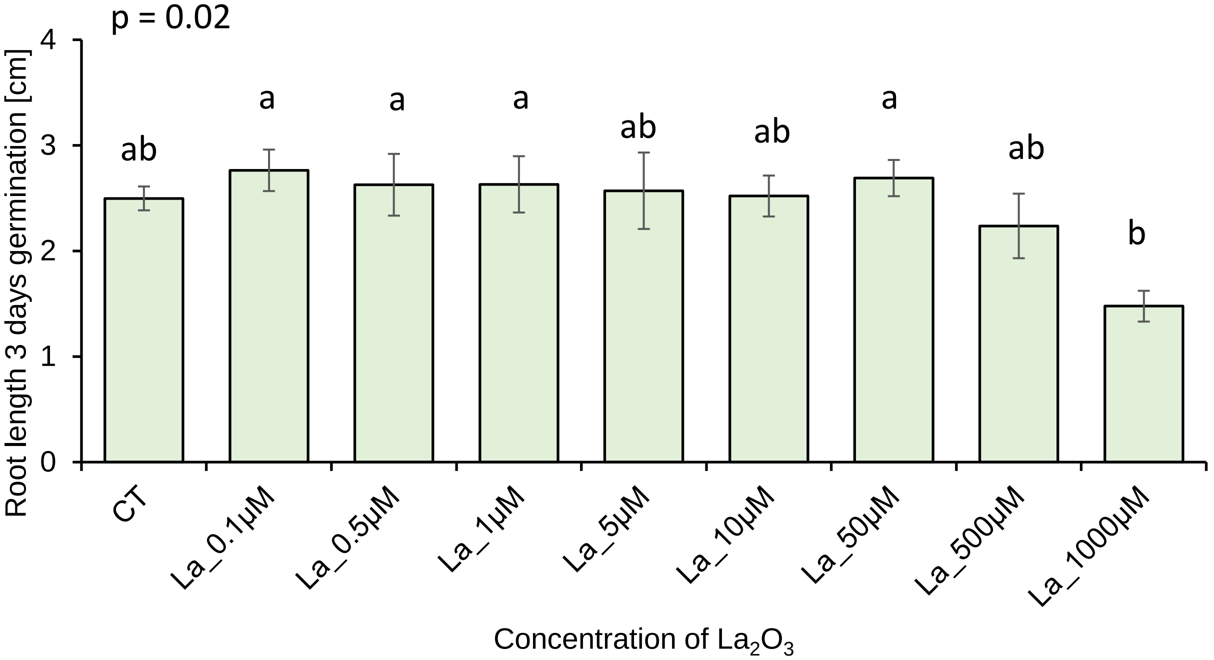

B

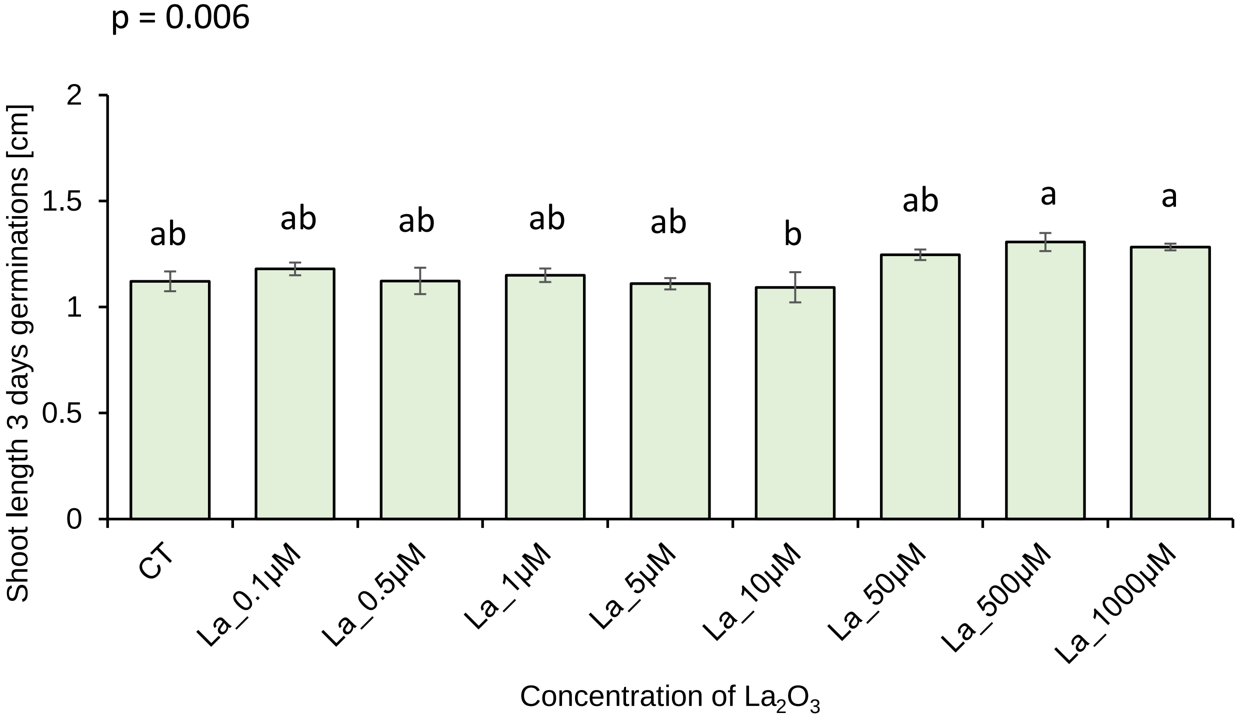

Supplement: Supplementary file 1 [file plants-14-00692-s001.zip › Supplementary Figure S2.pdf]
