# Supplementary material for: The Rare Earth Element Lanthanum (La) Accumulates in Brassica rapa L. and Affects the Plant Metabolism and Mineral Nutrition
Source: Plants (Basel). 2025 Feb 24;14(5):692. doi: 10.3390/plants14050692 (PMC11901600; doi:10.3390/plants14050692)
Supplement: Supplementary file 1 [file plants-14-00692-s001.zip › Supplementary Table S1.pdf]

Supplementary Table S1. Quantitative and qualitative determination of flavonoids extracted from *B. rapa* leaves of plants exposed to increasing La concentrations. Data are expressed as mg g-1 fr.wt (standard deviation).

Compound	Control (no La)	low La (1 μM)	medium La (1 mM)	high La (10 mM)
Apigenin-3-O-glucoside	0.65 (0.07)	0.64 (0.10)	0.64 (0.09)	0.83 (0.09)
Catechin	1.30 (0.14)	1.74 (0.361)	1.43 (0.33)	1.01 (0.10)
Catechin-3-O-arabinoside	0.11 (0.01)	0.20 (0.01)	0.24 (0.03)	0.38 (0.05)
Catechin-3-O-glucoside	0.47 (0.03)	0.94 (0.11)	0.93 (0.13)	1.04 (0.28)
Catechin-3-O-rutinoside	0.40 (0.04)	0.43 (0.04)	0.59 (0.04)	0.45 (0.06)
Dihidrokaemfero-3-O-rutinoside	0.90 (0.10)	0.87 (0.07)	0.80 (0.16)	0.95 (0.08)
Dihydrokaempferol-3- arabinoside	0.57 (0.06)	0.46 (0.05)	0.66 (0.10)	0.82 (0.11)
Dihydrokaempferol- sambubioside	0.53 (0.06)	0.69 (0.12)	0.92 (0.14)	0.57 (0.08)
dihydromercetin-diglucoside	0.25 (0.04)	0.25 (0.02)	1.23 (0.75)	0.18 (0.01)
Dihydromyricetin-3-O-glucoside	3.19 (0.66)	2.48 (0.15)	3.5 (0.67)	2.50 (0.36)
Dihydroquercetin-3-O-rutinoside	0.51 (0.087)	0.54 (0.07)	0.38 (0.03)	0.45 (0.04)
dihydroquercetin-3-O- sophoroside	1.18 (0.13)	1.35 (0.12)	1.42 (0.25)	1.64 (0.08)
Isohamentin-3-O-glucoside	7.69 (1.22)	10.29 (1.17)	10.27 (1.47)	11.21 (2.14)
Isorhamnetin-3-O-rutinoside	0.51 (0.04)	0.35 (0.05)	0.54 (0.12)	0.92 (0.17)
Kaempferol	0.45 (0.036)	1.03 (0.12)	0.88 (0.13)	1.04 (0.28)
Kaempferol-3-glucorinide	5.83 (1.25)	6.42 (0.90)	7.63 (0.94)	9.07 (1.63)
Kaempferol-3-O-glucoside	4.09 (0.49)	4.43 (0.51)	4.30 (0.71)	5.14 (0.46)

**Compound**	**Control (no La)**	**low La (1 μM)**	**medium La (1 mM)**	**high La (10 mM)**
Kaempferol-3-O-rhamnoside	3.78 (0.33)	5.07 (0.95)	4.11 (0.77)	3.22 (0.20)
Kaempferol-3-O-rutinoside	0.11 (0.01)	0.14 (0.01)	0.26 (0.01)	0.18 (0.02)
Kaempferol-7-sophoroside	0.52 (0.08)	0.47 (0.05)	0.42 (0.06)	0.44 (0.02)
Myricetin	3.91 (0.52)	4.28 (0.52)	4.16 (0.72)	4.63 (0.53)
Myricetin-3-O-glucoside	5.62 (0.96)	7.62 (0.86)	5.33 (0.81)	5.92 (1.00)
Naringenin-3-O-sambubioside	1.64 (0.28)	1.46 (0.31)	1.00 (0.23)	0.79 (0.10)
Quercetin	3.09 (0.33)	4.46 (1.02)	3.52 (0.86)	2.3 (0.22)
Quercetin-3-O-arabinoside	1.57 (0.15)	1.78 (0.36)	1.62 (0.32)	1.22 (0.10)
Quercetin-3-O-glucoside	2.72 (0.44)	2.78 (0.46)	3.43 (0.40)	4.50 (0.78)
Quercetin-3-O-rutinoside	0.57 (0.09)	0.66 (0.09)	0.39 (0.06)	0.46 (0.02)
Quercetin-3-O-sambubioside	0.91 (0.15)	1.45 (0.24)	0.95 (0.15)	0.92 (0.18)
Quercetin-3-O-sophoroside	2.65 (0.49)	3.84 (0.49)	2.69 (0.35)	2.49 (0.51)
