# Supplementary material for: The Rare Earth Element Lanthanum (La) Accumulates in Brassica rapa L. and Affects the Plant Metabolism and Mineral Nutrition
Source: Plants (Basel). 2025 Feb 24;14(5):692. doi: 10.3390/plants14050692 (PMC11901600; doi:10.3390/plants14050692)
Supplement: Supplementary file 1 [file plants-14-00692-s001.zip › Supplementary Table S2.pdf]

Supplementary Table S2. Fatty acid composition of *B. rapa* leaves exposed to increasing La concentrations. Values are expressed as mg g^{-1} fr.wt

Compound	Control (no La)	low La (1 μM)	medium La (1 mM)	high La (10 mM)
C16:0	9.36 ± 0.87	9.81 ± 1.20	9.08 ± 0.69	8.91 ± 0.89
C16:1cis	1.21 ± 0.06	1.36 ± 0.10	1.33 ± 0.11	1.25 ± 0.23
C16:1trans	12.58 ± 0.64	13.40 ± 1.38	13.67 ± 1.92	13.53 ± 1.48
C18:0	1.51 ± 0.14	1.51 ± 0.08	1.67 ± 0.52	1.67 ± 0.14
C18:2	8.54 ± 0.36	8.81 ± 0.82	9.08 ± 1.24	9.50 ± 0.71
C18:3	58.05 ± 1.56	57.43 ± 0.96	57.65 ± 1.11	57.38 ± 2.65
C20:0	2.60 ± 0.31	2.66 ± 0.18	3.31 ± 0.42	2.53 ± 0.18
C22:0	2.12 ± 0.42	2.79 ± 0.39	2.36 ± 0.06	2.43 ± 0.47
