# Supplementary material for: The Rare Earth Element Lanthanum (La) Accumulates in Brassica rapa L. and Affects the Plant Metabolism and Mineral Nutrition
Source: Plants (Basel). 2025 Feb 24;14(5):692. doi: 10.3390/plants14050692 (PMC11901600; doi:10.3390/plants14050692)
Supplement: Supplementary file 1 [file plants-14-00692-s001.zip › Supplementary Table S3.pdf]

Supplementary Table S3. Expression (Log2 fold change) of Selected Genes in *Brassica rapa* After 42 Days of Exposure to La. The Log2 (fold in change) means comparative gene expression levels between La treatment and control. *ns: $p > 0.05$; .: $0.05 < p < 0,1$; *: $p < 0.05$, **: $p < 0.01$, ***: $p < 0.001$. Different shade colors correspond to the heatmap colors of Figure 6*

**Category**	**Gene Name**	**Gene Locus* (NCBI)**	**Function/Description**	**Lanthanum concentration**		
				**low La (1 μM)**	**medium La (1 mM)**	**high La (10 mM)**
Carotenoids	NCED	LOC103870025	9-cis-epoxycarotenoid dioxygenase NCED3, chloroplastic) ABA synthesys	$-0.99 \pm 0.029^{***}$	$-0.91 \pm 0.036^{***}$	-0.99 ± 0.042
Carotenoids	LCYE	LOC103856778	Lycopene epsilon cyclase	$-0.21 \pm 0.015^{\bullet}$	$-0.32 \pm 0.141^{*}$	-0.29 ± 0.013
Carotenoids		LOC103863442	beta-carotene 3-hydroxylase 1, chloroplastic	$-0.62 \pm 0.004^{***}$	$-0.77 \pm 0.013^{***}$	$-0.56 \pm 0.010^{***}$
Carotenoids	Zep1	LOC103829545	zeaxanthin epoxidase, chloroplastic	0.36 ± 0.102^{ns}	$-0.58 \pm 0.102^{\cdot}$	0.22 ± 0.22^{ns}
Carotenoids	PSY	LOC103846262	phytoene synthase	$-0.23 \pm 0.004^{***}$	$-0.38 \pm 0.010^{***}$	-0.08 ± 0.027^{ns}
Carotenoids	PDS	LOC103835193	15-cis-phytoene desaturase, chloroplastic/chromoplastic	0.02 ± 0.005^{ns}	-0.04 ± 0.02^{ns}	$0.12 \pm 0.026^{*}$
Chlorophyll	Chlorophyll	LOC103841124	Chlorophyll synthase	$0.62 \pm 0.008^{***}$	$0.22 \pm 0.026^{**}$	$0.35 \pm 0.013^{***}$
Chlorophyll	RCCR	LOC103862886	Chloroph. Reduct, red chlorophyll catabolite reductase	$-0.70 \pm 0.013^{***}$	$-0.64 \pm 0.004^{***}$	$-0.46 \pm 0.014^{***}$
Phenolic Compounds	BrPal	LOC103867229	phenylalanine ammonia-lyase 1	$-0.44 \pm 0.028^{**}$	$-0.34 \pm 0.046^{*}$	-0.07 ± 0.049^{ns}
Phenolic Compounds	CHS-BR-2	LOC103854548	chalcone synthase	$-0.65 \pm 0.035^{**}$	$-0.62 \pm 0.036^{**}$	$-0.40 \pm 0.043^{**}$
Phenolic Compounds	CHI1	LOC103841428	chalcone--flavanone isomerase	$-0.20 \pm 0.008^{*}$	-0.09 ± 0.043^{ns}	$0.12 \pm 0.027^{*}$
Phenolic Compounds	FLS	LOC103847177	flavonol synthase/flavanone 3-hydroxylase	0.07 ± 0.002^{ns}	-0.02 ± 0.028^{ns}	$0.25 \pm 0.03^{*}$

Category	Gene Name	Gene Locus* (NCBI)	Function/Description	Lanthanum concentration		
				low (32.8ppb)	medium (32.58 ppm)	high (325.8 ppm)
Phenolic Compounds	ANS	LOC103860424	leucoanthocyanidin dioxygenase	-0.91 ± 0.032***	-0.67 ± 0.041***	-0.13 ± 0.03*
Phenolic Compounds	PAP1	LOC103858647	plastid lipid-associated protein 1, chloroplastic-like	0.16 ± 0.004***	0.15 ± 0.004***	0.16 ± 0.005***
Phenolic Compounds		LOC103847177	Flavanone 3 hydroxylase	-0.07 ± 0.005^{ns}	-0.19 ±0.053^{ns}	0.06 ± 0.06^{ns}
Phenolic Compounds		LOC103873769	putative inactive flavonol synthase 2	-0.15 ± 0.009^{ns}	-0.15 ± 0.084^{ns}	0.03 ± 0.071^{ns}
Ion Transporters	SOS	LOC103839460	Salt Overly Sensitive, sodium/hydrogen exchanger 7	0.16 ± 0.005*	0.02 ± 0.027^{ns}	0.20 ± 0.031**
Ion Transporters	ALMT13	LOC103853727	Aluminum-activated malate transporter 13	0.25 ± 0.007**	-0.16 ± 0.023*	0.34 ± 0.062**
Ion Transporters	HMA2	LOC103852939	Cadmium/Zinc-transporting ATPase HMA2	0.03 ± 0.002^{ns}	-0.10 ± 0.048^{ns}	0.15 ± 0.023**
Ion Transporters	ZntB	LOC103828536	Zinc transport protein ZntB	-0.03 ± 0.0002^{ns}	-0.12 ± 0.029^{ns}	0.07 ± 0.063^{ns}
Ion Transporters	PAA1	LOC103846231	Copper-transporting ATPase PAA1, chloroplastic	-0.52 ± 0.001$^{.}$	-0.12 ± 0.032$^{.}$	0.07 ± 0.018*
Ion Transporters	PDR2	LOC103865590	Manganese-transporting ATPase PDR2	0.000048 ± 0.000002^{ns}	-0.30 ± 0.017***	-0.045 ± 0.027^{ns}
Ion Transporters	Nramp1	LOC103832453	Metal transporter Nramp1	-0.56 ± 0.050^{ns}	-0.62 ± 0.072*	-0.47 ± 0.076$^{.}$
Ion Transporters	MOT1	LOC103853020	SULTR transporters Mo uptake (MOT1) molybdate transporter 1	-0.57 ± 0.026***	-0.69 ± 0.044**	-0.25 ± 0.030**
