# Supplementary material for: The Rare Earth Element Lanthanum (La) Accumulates in Brassica rapa L. and Affects the Plant Metabolism and Mineral Nutrition
Source: Plants (Basel). 2025 Feb 24;14(5):692. doi: 10.3390/plants14050692 (PMC11901600; doi:10.3390/plants14050692)
Supplement: Supplementary file 1 [file plants-14-00692-s001.zip › Supplementary Table S4.pdf]

Supplementary Table S4. Primers for genes used in this work

Categories	Gene Names	Gene Locus* (NCBI)	Functions/Description	Forward	Reverse
Carotenoids	NCED	LOC103870025	9-cis-epoxycarotenoid dioxygenase NCED3, chloroplastic ABA synthesys	ACGGGCAGTTAGAATCCACA	AGCTTGAACACGACTTGCTG
Carotenoids	LCYE	LOC103856778	Lycopene epsilon cyclase	TGAGGGAGGATTACGCTGAC	CAAAGCACCACCATCACCAA
Carotenoids		LOC103863442	beta-carotene 3-hydroxylase 1, chloroplastic	CCTAACATTCAAGCCGCTCC	TCGTGTCCAGAGAGCTTGTT
Carotenoids	Zep1	LOC103829545	zeaxanthin epoxidase, chloroplastic	TCGGAGGTTTAGTGTTCGCT	GATACATCCCGCCTCCATGA
Carotenoids	PSY	LOC103846262	phytoene synthase	TGGGTTGGTAAGGGCTGTAG	GCTCGAAGACACAACACTCC
Carotenoids	PDS	LOC103835193	15-cis-phytoene desaturase, chloroplastic/chromoplastic	TGTTGAGGCTCGAGATGGTT	CCTCATCTGTCACCCGATCA
Chlorophyll	Chlorophyll	LOC103841124	Chlorophyll synthase	GGCATATTCGTGACGGCATT	AGCGATCTCAGTTTCACCCA
Chlorophyll	RCCR	LOC103862886	Chloroph. Reduct, red chlorophyll catabolite reductase	AGACTCACCGTTCAAGCTCA	AAGGAGTTGAGAGTGGAGGC
Phenolic Compounds	BrPal	LOC103867229	phenylalanine ammonia-lyase 1	GAAGTGATCCGTTACGCCAC	CGGAGAACTGAGCGAACATG
Phenolic Compounds	CHS-BR-2	LOC103854548	chalcone synthase	TACAATGGTGATGGGTGCCT	TGAGGTCGGTCATGTGTTCA
Phenolic Compounds	CHI1	LOC103841428	chalcone--flavanone isomerase	GCTTCCTCCAACCCTCTCTT	ACGGAAGAAAGGGACGGATT
Phenolic Compounds	FLS	LOC103847177	flavonol synthase/flavanone 3-hydroxylase	GAAGAGTACGCGTTGCATGT	CCCAAAGCTAAATCCGGTCG
Phenolic Compounds	ANS	LOC103860424	leucoanthocyanidin dioxygenase	CCGTCCAAAAGAAGAGCTCG	TCCTGACTTCTTCACACGCT
Phenolic Compounds	PAP1	LOC103858647	lastid lipid-associated protein 1, chloroplastic-like	GGATTGAACTCATCGGTGGC	GGAGCTGGGTTAGGGTTCTT

Phenolic Compounds		LOC103847177	Flavanone 3 hydroxylase	GAAGAGTACGCGTTGCATGT	CCCAAAGCTAAATCCGGTCG
Phenolic Compounds		LOC103873769	putative inactive flavonol synthase 2	AAGACCAGCACACATCTCCA	AGCCTTCGAAGTCTTGGGAA
Ion Transporters	SOS	LOC103839460	Salt Overly Sensitive, sodium/hydrogen exchanger 7	CCAAATGCTCCGGTGTTGAA	TTGTACCATCTCCTGCTGCA
Ion Transporters	ALMT13	LOC103853727	Aluminum-activated malate transporter 13	CGGTCGGTTCAATCTCCAAC	GGAGTTTTCGGAGGCGTTAC
Ion Transporters		LOC103852939	Cadmium/Zinc-transporting ATPase HMA2	TGTTGTTGCTTCGGGATGTG	CCCGTTTCTTGTCACAGCAA
Ion Transporters		LOC103828536	Zinc transport protein ZntB	GGATAGTTGGCAAGCTGAGC	TCCTCCAACGTTCATCCCAA
Ion Transporters		LOC103846231	Copper-transporting ATPase PAA1, chloroplastic	GGTGGGCATTCGGATACAAC	CAGTCAATGGCAGCAACACT
Ion Transporters		LOC103865590	Manganese-transporting ATPase PDR2	TTCCTCTCTCTCCTCGGACA	ATGTCGCAACCTGAAGCATC
Ion Transporters		LOC103832453	Metal transporter Nramp1	ACTTCTTGCTTCCGGACAGA	CCAGCTGAACCACCAATGAG
Ion Transporters	MOT1	LOC103853020	SULTR transporters Mo uptake (MOT1) molybdate transporter 1	CCTCCCGTAGTTCCTCATCC	GGGTTGGTGTTGGGAAGTTC
Reference gene	GAPDH	LOC103848938	Glyceraldehyde-3-phosphate dehydrogenase	TTCCCACCGTTGATGTCTCA	CACGACACAAGCTTCACGAA

*source https://www.ncbi.nlm.nih.gov
