# Supplementary material for: The Rare Earth Element Lanthanum (La) Accumulates in Brassica rapa L. and Affects the Plant Metabolism and Mineral Nutrition
Source: Plants (Basel). 2025 Feb 24;14(5):692. doi: 10.3390/plants14050692 (PMC11901600; doi:10.3390/plants14050692)
Supplement: Supplementary file 1 [file plants-14-00692-s001.zip › Supplementary Table S5.pdf]

Supplementary Table S5. Raw data from ICP-MS analyses (μg/gDW). Different letters indicate a statistically significant difference ($P < 0.05$).

Mineral element	Control	La_low	La_Medium	La_High
Al	43.47 ± 1.06[a]	25.38 ± 1.20[b]	23.55 ± 0.46[b]	20.19 ± 2.06[b]
Cr	2.16 ± 0.33[a]	1.65 ± 0.09[ab]	1.21 ± 0.06[b]	1.26 ± 0.20[b]
Mo	11.02 ± 1.76	11.21 ± 1.36	7.21 ± 1.67	6.49 ± 0.53
Co	0.08 ± 0.00[a]	0.05 ± 0.00[b]	0.05 ± 0.01[b]	0.05 ± 0.00[b]
Ni	1.61± 0.03[a]	1.31 ± 0.09[ab]	1.08 ± 0.15[b]	1.01 ± 0.13[b]
P	5295.62 ± 184.67[b]	4261.98 ± 197.06[c]	6529.90 ± 323.36[a]	3414.64 ± 24.18[c]
Fe	95.82 ± 2.00[a]	97.45 ± 4.30[a]	84.73 ± 1.80[ab]	76.15 ± 3.49[b]
Se	0.43 ± 0.04[ab]	0.36 ± 0.02[ab]	0.48 ±0.03[a]	0.35 ± 0.03[b]
Na	9205.68 ± 664.82	7938.56 ± 957.88	6596.64 ± 744.51	8052.69 ± 305.38
Ca	35131.57 ± 1373.48[ab]	30270.95 ± 871.46[b]	37086.88 ± 1097.65[a]	30779.40 ± 1267.70[b]
Mg	6123.91 ± 223.28[a]	5498.87 ± 152.08[b]	6059.13 ± 130.48[a]	5489.21 ± 50.40[b]
Cu	4.71 ± 0.21	4.83 ± 0.04	4.53 ± 0.18	4.22 ± 0.08
Cd	0.49 ± 0.03	0.40 ± 0.03	0.49 ± 0.04	0.46 ± 0.03
Zn	138.95 ± 9.30	141.71 ± 8.78	125.34 ± 5.98	139.70 ± 0.92
Pb	0.35 ± 0.02	0.36 ± 0.03	0.33 ± 0.02	0.38 ± 0.05
As	0.36 ± 0.05	0.38 ± 0.05	0.29 ± 0.03	0.39 ± 0.02
Mn	172.10 ± 5.81	186.06 ± 8.17	211.34 ± 13.67	198.39 ± 7.11
K	31139.64 ± 2260.13[ab]	26152.94 ± 446.88[b]	27902.51 ± 2680.52[b]	37237.08 ± 1058.14[a]
Tl	0.12 ± 0.02	0.11 ± 0.01	0.12 ± 0.01	0.15 ± 0.00
La	0.23 ± 0.07[b]	0.12 ± 0.01[b]	0.38 ± 0.04[b]	9.36 ± 1.41[a]
